# Parents’ Perception of the Role of Sports in Supporting Psychological and Social Development in Children with Specific Learning Disorders

**DOI:** 10.3390/children11121493

**Published:** 2024-12-07

**Authors:** Fernanda Borges-Silva, Aarón Manzanares, Noelia González-Gálvez, Giuseppe Zanzurino, Gabriele Cordovani, Domenico Cherubini, María T. Morales-Belando

**Affiliations:** 1 Facultad de Deporte, UCAM—Universidad Católica de Murcia, 30107 Guadalupe, Spain; bsfernanda@ucam.edu (F.B.-S.); amanzanares@ucam.edu (A.M.); ngonzalez@ucam.edu (N.G.-G.); dcherubini@ucam.edu (D.C.); 2 AID—Italian Dyslexia Association, 40121 Bologna, Italy; zanzurinogiuseppe@gmail.com (G.Z.); gabri.cordovani@gmail.com (G.C.)

**Keywords:** dyslexia, dyscalculia, sports practice, well-being, self-esteem

## Abstract

Background: This study aims to examine parents’ perceptions of how coach support influences the satisfaction of basic psychological needs (autonomy, competence, and relatedness) and its subsequent impact on the self-esteem and overall well-being of children with Specific Learning Disorders (SLDs) through participation in sports. Methods: The sample consisted of 1146 parents of children and young people diagnosed with SLDs from several European countries. The Coach Support Scale (COS), the Basic Psychological Needs Satisfaction Scale (BPNS), the Rosenberg Self-Esteem Scale (RSES), and the Sport Impact Scale (SIS) were used. Descriptive, reliability, Gaussian distribution, and ANOVA analyses were conducted. Results: The results show that increased sports participation is associated with higher perceptions of coach support, autonomy, competence, psychological need satisfaction, self-esteem, relatedness, and the positive impact of sports on the lives of children and adolescents with SLDs. Children and teenagers who engage in individual and team sports exhibit greater coach support, autonomy, competence, relatedness, BPNS, and self-esteem compared to those who participate in only one type of sport, with *p*-values ranging from 0.004 to 0.050. Conclusions: In conclusion, participation in sports seems to benefit the self-esteem and well-being of children and young people with SLDs. The practical applications of this study highlight the importance of specialized coach training to address the psychological needs of children with SLDs, the development of balanced sports programs that integrate individual and team activities to optimize benefits, the encouragement of parental involvement to enhance positive experiences, and the implementation of policies that support inclusive and adaptive sports practices.

## 1. Introduction

Children with Specific Learning Disorders (SLDs) experience significant difficulties in one or more areas of learning, such as reading, mathematics, or writing, which cannot be explained by factors like low IQ, lack of motivation, or cultural or linguistic barriers. These difficulties can have profound impacts on their self-esteem, academic performance, and emotional well-being [1,2], reducing their participation in school, home, and sports activities [3], and making them more prone to underestimating their abilities [4]. The evident academic challenges faced by children with SLDs often lead to social difficulties, as they may feel marginalized within the school environment due to their inability to achieve the same level as their peers in academic tasks. This situation fosters frustration, low self-esteem, and social isolation, resulting in reduced enjoyment and lower participation in daily activities [2,5,6]. These children also experience difficulties with emotional self-regulation, making them more vulnerable to anxiety, depression, and other emotional disorders [1].

Sports and physical activities provide a unique and potentially highly beneficial environment for children with SLDs. Sports can offer these children an opportunity to experience success outside the academic context, significantly boosting their self-esteem and self-confidence [7,8,9]. Additionally, sports can enhance cognitive function and motor development in children with SLDs, optimizing their overall learning and development [10]. Individual and team sports have different impacts on child development. Team sports are associated with lower perceived stress, reduced sports anxiety, and higher motivation compared to individual sports [11]. However, individual sports tend to generate fewer antisocial behaviors than team sports [12].

Previous studies show how participation in sports activities can improve children’s self-efficacy, especially in those facing cognitive barriers. The ability to achieve goals in a sports environment enhances the perception of competence, a crucial factor for positive emotional development [5]. Additionally, sports promote significant improvements in emotional regulation, as children must learn to manage frustration, competition, and mistakes, which strengthens their ability to cope with stressful situations both in academic settings and in daily life [13,14].

Social relationships can positively influence the self-esteem of adolescents with learning disorders [15]. Sports encourage interaction among children, promoting the development of social and communication skills. Children with SLDs, who often struggle to form relationships in the classroom due to their academic performance, may find in sports an opportunity to connect with their peers in a more inclusive context. Teamwork and collaboration are essential in sports, allowing children with SLDs to learn social skills that might not be adequately developed in the classroom [7,14,16,17,18].

For example, Bailey and Dismore [5] highlight how sports activities not only improve children’s self-esteem but also provide opportunities for them to learn teamwork, manage emotions, and collaborate with others—factors that contribute to their overall well-being. Additionally, studies by Fraser-Thomas et al. [7] suggest that participation in sports fosters resilience in children, as it allows them to experience and overcome setbacks, which can be a key factor in their emotional and psychological development.

The satisfaction of basic psychological needs (BPNs—competence, autonomy, and relatedness) in sports and physical education is associated with greater life satisfaction and intrinsic motivation among adolescents [19,20]. Factors such as years of practice, training frequency, and competitive level are linked to the fulfillment of these needs, with more experience and training enhancing competence and autonomy [21]. These findings highlight the importance of supporting basic psychological needs in sports contexts to improve motivation and overall well-being in young athletes. Support for BPNs and low pressure are associated with higher motivation and less boredom in young athletes [22]. Autonomy support significantly predicts satisfaction with BPNs, which in turn influences intrinsic motivation and the intention to remain physically active [20].

It is important to highlight that the way teachers perceive and approach these children also plays a crucial role in their school experience. The lack of specific training to identify and address the needs of children with SLDs, particularly among physical education teachers and other sports specialists, can result in the exclusion of these children from activities that could otherwise be beneficial. In many cases, children with SLDs do not receive the proper support to engage in sports or recreational activities that promote their physical and emotional well-being [13].

Specifically, a study found that a self-esteem enhancement program for children aged 8 to 11 with SLDs was effective in improving various aspects of self-esteem [23]. Parents’ perceptions of teachers’ attitudes are crucial for assessing the child’s immediate environment, and a better teacher–child relationship leads to greater parental involvement in their children’s sports activities [24]. A coach and parent training program in social skills and problem solving showed positive results in enhancing the educational influence of parents on their children through the work of the coaches [25]. Therefore, sports programs for children with SLDs should support the development of their self-esteem [26], given the important role that coaches play in fostering the self-esteem of children with SLDs.

It is important to highlight the significance of creating inclusive sports environments for children with SLDs [27]. Strategies to promote integration include raising awareness, adapting communication methods, and providing practical support [28]. Factors that influence successful integration include attitudes, behavior management, and individuals’ previous experiences in sports and inclusive settings [28]. Sports can enhance motor skills, self-esteem, and social inclusion in children with SLDs, addressing motor challenges and fostering development [29]. Coaches play a key role in creating inclusive environments for children and adolescents with learning disorders and neurological developmental disorders by adapting communication styles, modifying training settings, and critically reflecting on personal biases [30]. While there is an increase in the participation of children with special needs in sports, coaches and teachers often lack adequate knowledge about the challenges faced by children with intellectual and learning disorders [31]. Therefore, it is essential to improve the professional skills of coaches and educators to better support and promote the development of children with SLDs through sports.

Despite the evidence on the benefits of sports for children with SLDs, there are significant gaps in knowledge that need to be addressed. Most existing studies do not directly compare the effects of sports with other more traditional interventions, such as psychopedagogical support or academic reinforcement programs. While the value of sports is acknowledged, there is a lack of systematic and comparative evaluation of its impact in relation to other therapeutic and educational approaches [32]. 

This study aims to examine parents’ perceptions of how coach support influences the satisfaction of basic psychological needs (autonomy, competence, and relatedness) and its subsequent impact on the self-esteem and overall well-being of children with SLDs through participation in sports.

## 2. Materials and Methods

### 2.1. Study Design

This is a cross-sectional study. This study was conducted in three European countries: Italy, Spain, and Ireland. The participants were informed about the project, and an informed consent was obtained. All data collected were treated confidentially and anonymously. This study followed the Strobe Statement. The main author’s University Research Ethics Committee approved the study, which was performed following the Helsinki Declaration.

### 2.2. Participants

The study sample included 1146 parents of children and adolescents with SLDs, of whom 94.4% were mothers and 5.6% were fathers. The children, aged between 7 and 20 years, were diagnosed with dyslexia, dysgraphia, dysorthography, and dyscalculia, with 61.6% being boys and 38.4% girls. The participants came from three countries: Italy (*n* = 578), Spain (*n* = 115), and Ireland (*n* = 453). The inclusion criteria were as follows: (a) having a child or young adult with an SLD; (b) signing the informed consent. The exclusion criteria were the following: that the child had been diagnosed with another pathology that would affect their development and practice of physical activity, understanding, or functioning in daily life. The sample size and power calculations were performed using Rstudio 3.15.0 software. The significance level was set at α = 0.05 and a power of 95% (1 − β = 0.95) was used. Standard deviation was used, according to the standard deviation for the Satisfaction of Basic Psychological Needs Scale. The sample provided a power of 95% if found between and within a variance of 0.052 points in the scale. 

### 2.3. Measuring Instruments

Below are the scales that have been adapted to different contexts, as well as the different constructs that we have measured and used in the research through these scales. 

Coach Support Scale (COS-Parents): A new questionnaire was composed to measure the level of support from the coach towards children/youth with SLD. A very high reliability has emerged. It was therefore possible to calculate the variable and perform the analysis of the Gaussian distribution α = 0.95, demonstrating a good fit of the statistics (M = 21.8 SD = 5.2). Examples: “In my opinion, my son/daughter, in relation to his/her coach, feels to be… understood… encouraged to trust in his/her abilities… free to express his/her feelings”. This allowed us to explore the levels of impact of sport on children/youth according to some socio-demographic characteristics.

Satisfaction of Psychological Needs: The Basic Psychological Needs Scale (BPNS), created by Deci and Ryan [33], measures the satisfaction of three psychological needs: autonomy (4 items), competence (4 items), and relatedness (4 items) (Likert 1–4). The reliability of the subscales reported the following values of Cronbach’s alpha: autonomy, 0.78; competence, 0.83; relatedness, 0.83. It was therefore possible to calculate the variable and to perform the analysis of the Gaussian distribution α = 0.92, demonstrating a good fit of the statistics (M = 37.52 SD = 7.27). Examples: “During training, my son (daughter) feels… autonomous to perform the exercises during the training… feels able to perform the exercises well… likes spending time with his/her coach”. This scale allowed us to explore the levels of impact of sport on children/youth psychological needs according to some socio-demographic characteristics.

Rosenberg Self-Esteem Scale (RSES): Originally intended as a measure of self-esteem for adolescents, Rosenberg’s Self-Esteem Scale is probably the most widely used measure of self-esteem [34]. The scale is composed of 10 items. A very high reliability has emerged. It was therefore possible to calculate the variable and to perform the analysis of the Gaussian distribution α = 0.89, demonstrating a good fit of the statistics (M = 28.3 SD = 6.07). Examples: “On the whole, my son/daughter is satisfied with him/himself… He/she feels to have a number of good qualities… Sometimes he/she feels completely useless”. This scale allowed us to explore the levels of impact of sport on children/youth self-esteem according to some socio-demographic characteristics.

Sport Impact Scale (SIS-Parents): From the analysis of the reliability of the scale, composed of 10 items, a high internal consistency has emerged. It was therefore possible to calculate the variable and carry out the analysis of the Gaussian distribution. The scale measures the psychophysical impact of sport on children/youth α = 0.88, demonstrating a good fit of the statistics (M = 27.84 SD = 5.93). Examples: “Since my son (daughter) has been practising sport… is getting better and better at his/her sport… he/she is physically better… feels better about himself/herself… it is easier for him/her to study”. This allowed us to explore the levels of impact of sport on children/youth according to some socio-demographic characteristics.

### 2.4. Procedure

The research was conducted in collaboration with the national SLD associations from the three partner countries. Through these associations, information about the study was distributed to all participants. The survey was made available to anyone who wished to participate and completed the informed consent form. Once the SLD associations had obtained authorization to administer the questionnaires to the parents, the participants were informed about the completion of said questionnaires, as well as the anonymity of their responses, with participation being completely voluntary. The participants completed the questionnaires independently and in a calm and quiet environment, which encouraged relaxation and concentration. The instructions established before completing the questionnaires referred to the general objective of the study, to show greater interest in completing them, as well as the mechanics of completing them. Similarly, some terms that might be confusing were clarified and they were encouraged to fill out the questionnaires as honestly as possible, emphasizing their anonymity. The time required to complete the questionnaires was approximately 15 min, varying slightly.

### 2.5. Statistical Analysis 

In this section, we analysed descriptive data. To check the differences between sports levels and the differences between individual or team sports according to the parents’ perception of coach support, autonomy, competition, social relationships, basic needs psychological characteristics, self-esteem, and the impact of sport on the lives of children and young people with SLDs who practice sports, a different analysis of variance (ANOVA) was carried out.

## 3. Results

According to descriptive results, participants reported the following characteristics of their children: In response to the question “What kind of sport does he/she practice?”, 31% of the parents stated that their children play individual sports, 42.4% play team sports, and 26.6% play both types of sports; football, swimming, basketball, volleyball, and rugby were the disciplines most played by their children. In terms of level, 24% consider their children to be beginners, 37.7% intermediate, and 38.3% competitive. The frequency of the children’s training varied, with 2.4% training occasionally, 13.5% training once a week, 62.6% training several times a week, and 21.5% training every day. Reasons for playing sports according to the parents included enjoyment (74.6%), pleasure from movement (53.4%), socializing with peers (51.6%), health benefits (47.5%), and keeping fit (31.6%). On the other hand, according to parents, for the 44.6% of the children who do not participate in sport, a lack of time was cited as the main reason, with 30.8% of children not participating because they do not like sport. Regarding perceptions about sport, the following observations were made (“Since my son (daughter) has been practicing sport…”): a high percentage of those who played sport reported improvements in their sport performance (84%), physical fitness (88.7%), personal well-being (83%), and a reduction in school stress (51.8%). In addition, 69% indicated that their child spent less time playing video games or using mobile phones, and 74.4% reported that their child was happier in general. When parents were asked “Have you encountered dyslexia/SLD related problems in your child’s participation in sports?”, 57% of parents answered “no” compared to 43% who said “yes”. 

According to parents’ perceptions, statistically significant differences were observed across varying levels of sports participation (Table 1). As the level of sport participation increases, several factors increase correspondingly: perceived support from coaches, children’s autonomy, children’s competence, children’s relatedness, satisfaction of psychological needs, self-esteem, and the impact of sport on the lives of children and adolescents.

Statistically significant differences were found between individual and team sports. According to parents’ perception, individual sports have a greater impact on the perception of coach support (*p* = 0.004). Parents of children who practice both individual and team sports report higher levels of coach support (*p* = 0.028) and greater satisfaction with children’s psychological needs (*p* = 0.035), autonomy (*p* = 0.029), and relatedness (*p* = 0.041). No differences were found in the perception of the impact of sports on children’s lives (Table 2).

Statistically significant differences were found across several variables. For coach support, there was a significant difference between the perception of the parents in team sports (mean 21.2) and both individual and team sports (mean 22.2), with a *p*-value of 0.028. Additionally, a significant difference was observed between individual (mean 22.3) and team sports (mean 21.2), with a *p*-value of 0.004. Regarding parents’ perceptions of children’s autonomy, a significant difference emerged between those who engage in team sports (mean 12.2) and those who engage in both individual and team sports (mean 12.6) (*p* = 0.029).

Regarding parents’ perceptions of children’s competence, a significant difference was found between those participating in individual sports (mean 12.0) and those participating in both individual and team sports (mean 12.5) (*p* = 0.050). For parents’ perceptions of children’s relatedness, the difference between those participating in team sports (mean 12.8) and those participating in both individual and team sports (mean 13.2) was statistically significant (*p* = 0.041). In terms parents’ perceptions of children’s Basic Psychological Needs Satisfaction (BPNS), a significant difference was detected between those involved in team sports (mean 37.1) and those involved in both individual and team sports (mean 38.4), with a *p*-value of 0.035. Finally, for parents’ perceptions of children’s self-esteem, there was a significant difference between those involved in individual sports (mean 27.8) and those engaging in both individual and team sports (mean 29.0) (*p* = 0.044).

## 4. Discussion

The aim of this study was to investigate parents’ perceptions of coach support and the impact of sports practice on the development of satisfaction of psychological needs, self-esteem, and in the life of their children with SLDs. From the parents’ perspective, it is interesting to observe how they perceive the impact of sport according to their children’s levels of sport practice. The results found that, as levels of sport increase, there is an increase in the perception of support from the coach, satisfaction of psychological needs, self-esteem, and the positive impact of sport on young people’s lives. 

The perception of coach support is a crucial factor influencing children and young people’s motivation and well-being in sport. This is in line with previous studies that emphasize that emotional and social support from coaches can enhance the sport experience and engagement among young people [35]. This is especially relevant for children with learning difficulties, who can benefit greatly from an environment that validates their efforts and strengths. In addition, the satisfaction of psychological needs, including competence, autonomy, and relatedness to others, is intensified as young people engage in higher levels of sport. This is consistent with the Self-Determination Theory, which argues that satisfying these basic needs is fundamental to well-being and intrinsic motivation [36]. Increasing the level of sport could therefore promote a significant contribution to meeting the basic psychological needs of children and young people with SLDs, which is essential for their personal and social development. The improvement in self-esteem observed in the study is also in line with the literature, which indicates that sporting proficiency is related to better self-image and psychological well-being. According to a meta-analysis [37], young people who participate in physical activities tend to report higher levels of self-esteem and personal satisfaction, which is highly relevant for those with learning difficulties. The impact of sport on the lives of children and young people with SLDs, as evidenced by our results, suggests that increased involvement in sporting activities may be associated with benefits beyond the physical domain, including improvements in mental health, resilience, and social skills [38].

One of the most notable differences in this study is that the parents of children with SLDs who participate in individual sports perceive greater support from the coach. This finding may be related to the nature of individual sports, where the interaction between the coach and the child is more direct and personalized. In sports like swimming, athletics, or tennis, the coach has a clearer opportunity to focus on the individual needs and skills of each child, providing specific training and closer follow-up [29]. Coaches in individual sports tend to have a more prominent role as mentors and emotional support figures, which can strengthen the child’s perception of support [13,39,40].

This type of direct interaction can be particularly beneficial for children with SLDs, who often require a more personalized approach tailored to their emotional and psychological needs. In fact, some studies highlight that the one-on-one relationship between the child and the coach in individual sports can provide a platform for the child to develop self-control and self-confidence skills, which are fundamental for their self-esteem and overall well-being [1].

On the other hand, the results also reveal that parents of children with SLDs who participate in both individual and team sports perceive a greater satisfaction of the children’s psychological needs, including autonomy, relatedness, and competence. Previous studies have found that participation in team sports significantly improves children’s social skills [41], such as communication, cooperation, and conflict resolution, which can contribute to greater psychological well-being [5]. Children who participate in team sports develop a sense of group identity and learn to work together towards a common goal, which can also strengthen their self-esteem by experiencing shared achievement and collective reward [7].

Success in sports, whether individual or team-based, reinforces children’s perception of competence and ability, which in turn improves their self-esteem [13,42]. Self-efficacy, defined as the belief in one’s ability to achieve goals, is a key concept here. In individual sports, self-efficacy can be reinforced through personal achievements, while in team sports, it is reinforced through shared success and the sense of belonging to a group [5].

In addition, the perception of support from the coach, whether in individual or team sports, plays a crucial role in the development of self-esteem. As Fraser-Thomas et al. [7] note, a coach who provides positive feedback, validates the child’s efforts, and fosters an inclusive environment can significantly influence the child’s self-perception, improving their self-esteem. Autonomy is another key factor, as children who feel they have control over their participation in sports activities experience a greater sense of competence and self-esteem [43].

The fact that children who participate in both individual and team sports report greater satisfaction with their psychological needs may be linked to the complementary nature of these two types of sports. While team sports can promote social integration and collaboration, individual sports provide opportunities for the development of autonomy and self-confidence [32]. This combination may offer a more balanced approach, benefiting both personal self-efficacy and social skills, ultimately contributing to greater overall well-being in children with SLDs. This integrated approach could be more effective in meeting the psychological needs and enhancing the self-esteem of children with SLDs, key elements according to Self-Determination Theory [36].

Finally, on self-esteem, the results show that participation in individual sports is associated with a lower perception of self-esteem compared to those who participate in both types of sports. This may reflect the fact that, in individual sports, success or failure is more dependent on individual performance, which may affect self-esteem more directly. The literature, such as the research of Ullrich-French and Smith [44], indicates that participation in team sports may contribute to a sense of belonging and social support, factors that can raise self-esteem. However, it is important to note that no differences were found in parents’ perceptions of the impact of sport on the lives of young people with SLDs and the different forms of sport played. This finding suggests that, regardless of whether children and youth with SLDs participate in individual or team sports, they may experience similar developments in these critical areas. It is important to consider that parents’ perceptions may be influenced by several factors, including their own sport experiences and their understanding of child development. Therefore, future research should explore how these perceptions correlate with young people’s direct reports of their experience of sport.

## 5. Conclusions

This study examined parents’ perceptions of the role of coach support in meeting the basic psychological needs (autonomy, competence, and relatedness) of children with Specific Learning Disorders (SLDs), and its broader impact on their self-esteem and well-being through sports participation. The findings highlight the crucial role that sports involvement, especially in environments where coaches offer tailored support, plays in the psychological and social development of children with SLDs.

Key results indicate that higher levels of sports engagement are associated with greater satisfaction of psychological needs, higher self-esteem, and a stronger positive impact on children’s overall well-being. Individual sports appear to foster more direct and personalized coach support, while participation in both individual and team sports offers the highest satisfaction of autonomy, relatedness, and competence. Parents perceive that inclusive and adaptive coaching strategies significantly enhance the positive outcomes of sports participation for children with SLDs, aligning with the principles of Self-Determination Theory.

The findings highlight several practical implications, including the need for coaches working with children with SLDs to receive specialized training to better understand and support their unique psychological needs. This training should focus on fostering autonomy, providing constructive feedback, and creating inclusive environments. Sports programs for children with SLDs should aim to strike a balance between individual and team sports to maximize psychological benefits. Combining both types of sports offers diverse experiences that enhance autonomy and relatedness while also building competence. Parents should be encouraged to actively participate in sports-related activities and maintain regular communication with coaches to reinforce positive experiences and advocate for their children’s needs. Educational and sports organizations should implement policies that promote inclusive sports practices, ensuring access to adaptive training methods and resources for children with SLDs.

In conclusion, this study highlights the importance of supportive coaching and well-structured sports programs in fostering the psychological well-being and personal development of children with SLDs. Future research should explore how these findings can be applied across various cultural and sporting contexts to further enhance the benefits of sports participation for this population.

This study acknowledges several limitations that may have influenced the findings. While inclusion and exclusion criteria were clearly defined, other factors such as the participants’ socioeconomic status, the severity of their children’s Specific Learning Disorders (SLDs), and which parent completed the questionnaire were not controlled or analyzed. These variables could potentially impact both access to sports programs and the perceived effects of sports on psychological and social development. Future research should consider these factors to gain a more comprehensive understanding of the varied experiences and outcomes within this population.

## Figures and Tables

**Table 1:** Differences between levels of sport according to parents’ perception.

					Beginner—Intermediate		Beginner—Advanced		Intermediate—Advanced	
	Level of Practice	N	Mean	SD	*p*	Mean Dif	*p*	Mean Dif	*p*	Mean Dif
Coach support	Beginner	265	20.8	5.25	0.086	−0.848	**<0.001**	−1.94	**0.005**	−1.09
	Intermediate	446	21.6	5.23						
	Advanced (competitive)	448	22.7	5.04						
Children’s Autonomy	Beginner	265	11.2	2.71	**<0.001**	−1.06	**<0.001**	−1.982	**<0.001**	−0.925
	Intermediate	446	12.3	2.50						
	Advanced (competitive)	448	13.2	2.44						
Children’s Competence	Beginner	265	10.8	2.78	**<0.001**	1.36	**<0.001**	−2.42	**<0.001**	1.06
	Intermediate	446	12.1	2.48						
	Advanced (competitive)	448	13.2	2.34						
Children’s Relatedness	Beginner	265	12.2	2.64	**0.002**	−0.659	**<0.001**	−1.506	**<0.001**	−0.847
	Intermediate	446	12.8	2.55						
	Advanced (competitive)	448	13.7	2.42						
Children’s BPNS	Beginner	265	34.1	7.49	**<0.001**	−3.07	**<0.001**	−5.91	**<0.001**	−2.83
	Intermediate	446	37.2	6.89						
	Advanced (competitive)	448	40.0	6.51						
Children’s Self-esteem	Beginner	265	26.6	5.66	**0.002**	−1.56	**<0.001**	−2.79	**0.007**	−1.22
	Intermediate	446	28.2	6.17						
	Advanced (competitive)	448	29.4	6.04						
Children’s Sport impact	Beginner	265	24.9	5.63	**<0.001**	−2.73	**<0.001**	−4.85	**<0.001**	−2.12
	Intermediate	446	27.6	5.59						
	Advanced (competitive)	448	29.8	5.62						

**Table 2:** Differences between individual and team sports by parents’ perception.

					Individual—Team		Individual—Both		Team—Both	
	Type of Sport	N	Mean	SD	*p*	Mean Dif	*p*	Mean Dif	*p*	Mean Dif
Coach support	Individual sport	367	22.3	4.93	**0.004**	1.13	0.898	0.176	**0.028**	−0.957
	Team sport	502	21.2	5.45						
	Both ^1^	314	22.2	5.04						
Children’s Autonomy	Individual sport	367	12.4	2.53	0.464	0.214	0.378	−0.270	**0.029**	−0.484
	Team sport	502	12.2	2.71						
	Both	314	12.6	2.65						
Children’s Competence	Individual sport	367	12.0	2.54	0.800	−0.117	**0.050**	−0.48	0.142	−0.363
	Team sport	502	12.1	2.73						
	Both	314	12.5	2.73						
Children’s Relatedness	Individual sport	367	13.1	2.52	0.244	0.285	0.681	−0.166	**0.041**	−0.451
	Team sport	502	12.8	2.68						
	Both	314	13.2	2.52						
Children’s BPNS	Individual sport	367	37.4	6.96	0.723	0.382	0.229	−0.916	**0.035**	−1.298
	Team sport	502	37.1	7.48						
	Both	314	38.4	7.24						
Children’s Self-esteem	Individual sport	367	27.8	5.98	0.601	−0.401	**0.044**	−1.119	0.227	−0.718
	Team sport	502	28.2	5.98						
	Both	314	29.0	6.29						
Children’s Sport impact	Individual sport	367	27.7	5.91	0.976	−0.086	0.863	−0.236	0.934	−0.15
	Team sport	502	27.8	5.75						
	Both	314	28.0	6.24						

^1^ both: refers to parents of children who play individual and team sports simultaneously, for playing more than one sport.

## Data Availability

The original contributions presented in this study are included in the article. Further inquiries can be directed to the corresponding authors.
